# Rupture of the ulnar collateral ligament of the thumb – a review

**DOI:** 10.1186/1865-1380-6-31

**Published:** 2013-08-12

**Authors:** Mandhkani Mahajan, Steven J Rhemrev

**Affiliations:** 1 Department of Surgury, Medisch Centrum Haaglanden, The Hague, The Netherlands

**Keywords:** Skier’s thumb, UCL, Hand injury, Ligament rupture, Sports injury

## Abstract

Skier’s thumb is a partial or complete rupture of the ulnar collateral ligament of the metacarpophalangeal joint of the thumb. It is an often-encountered injury and can lead to chronic pain and instability when diagnosed incorrectly. Knowledge of the anatomy and accurate physical examination are essential in the evaluation of a patient with skier’s thumb. This article provides a review of the relevant anatomy, the correct method of physical examination and the options for additional imaging and treatment with attention to possible pitfalls.

## Search strategy

The literature search was conducted on PubMed in the week of 5 November 2012. An additional search for updates on the literature was performed on 1 May 2013. No MeSH terms were available for this subject; therefore the following keywords were used: ‘skier’s thumb,’ ‘ulnar AND collateral AND ligament, NOT elbow,’ ‘UCL, NOT elbow’ and ‘gamekeeper’s thumb.’ Filters applied were studies in humans and publications in English. Recent articles were preferred; however due to the limited amount of information available about this subject, articles of older dates were also reviewed. Additional information concerning our own hospital was worked out by the primary author.

## Introduction

A partial or complete rupture of the ulnar collateral ligament of the metacarpophalangeal joint of the thumb, skier’s thumb, is an often-encountered problem. It concerns 86% of all injuries to the base of the thumb [1]. The estimated incidence in the US is approximately 200,000 patients per year [2]. The incidence in the Netherlands is not known. In the last four years, we have diagnosed approximately 85 patients in our own hospital.

Skier’s thumb is the result of a hyperabduction trauma of the thumb. It can occur with any fall on an outstretched hand when a thumb that is already in abduction receives an extra valgus stress. Skier’s thumb refers to the fact that this injury is often seen in skiers who fall while holding on to their ski poles. Despite renewed designs for ski poles, skier’s thumb remains a common injury. Prevalence of this injury during skiing varies from 7% [3] up to as high as 32% of all skiing injuries [4]. This makes it the most common injury of the upper extremities during skiing.

This type of injury is also seen in other sports, especially those that use a stick or ball, such as hockey or basketball. During a query in our own inner-city hospital, only 10% of the patients had skier’s thumb due to an injury acquired during skiing. Often, these patients also presented with a delay because their injury occurred during a holiday, and they waited until they came back home to see their own physician. A fall on the hand, usually from a bicycle or motorcycle (in which the thumb gets stuck behind the handlebars), is the most common cause of skier’s thumb in our hospital, seen in approximately 40 % of all patients. Another sport, especially soccer or fighting, was the cause in 30%.

In children, who still have an immature skeleton, hyperabduction trauma mostly leads to a Salter-Harris III avulsion of the UCL insertion and rarely to a true rupture of the UCL [5].

### Anatomy

**Figure 1:**
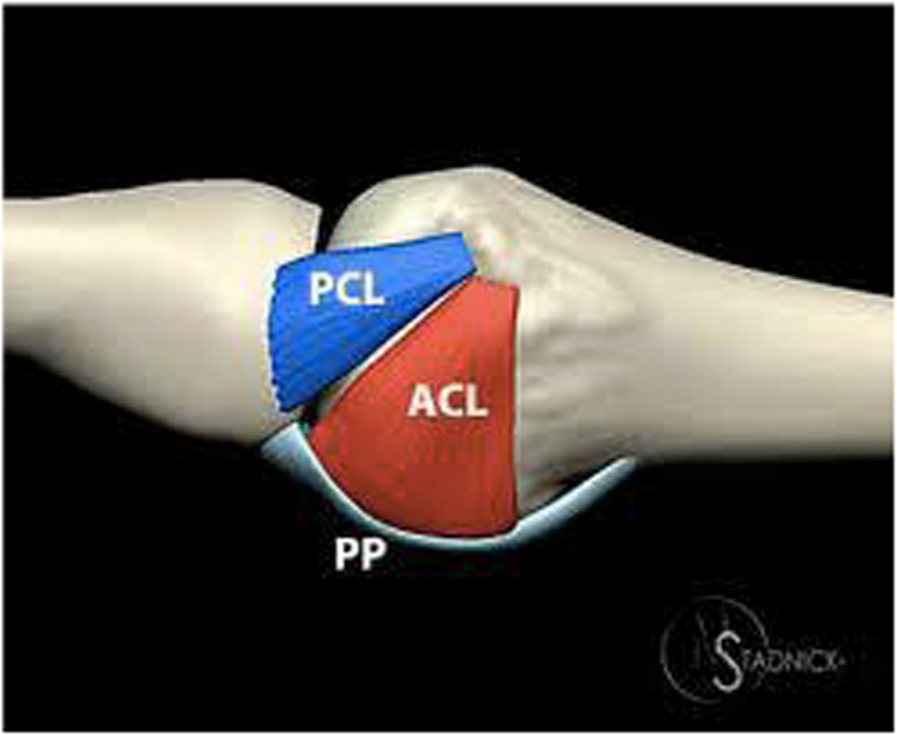
Anatomy of the ACL and PCL.

The ulnar collateral ligament is made up of two parts, the proper collateral ligament (PCL) and the accessory collateral ligament (ACL). The PCL has its origin proximal to the base of the head of the MCP-1 joint and its insertion on the volar side of the proximal phalanx. The ACL has its origin just palmar of the PCL and runs parallel to the PCL to its insertion on the proximal phalanx (Figure 1). Together they ensure the ulnar and volar stability of the base of the thumb. However, there are other components that also take part in creating stability in the joint. They can be divided into static and dynamic components. Next to the PCL and ACL, the shape of the joint, the dorsal capsule and the volar plate make up the static components.

**Figure 2:**
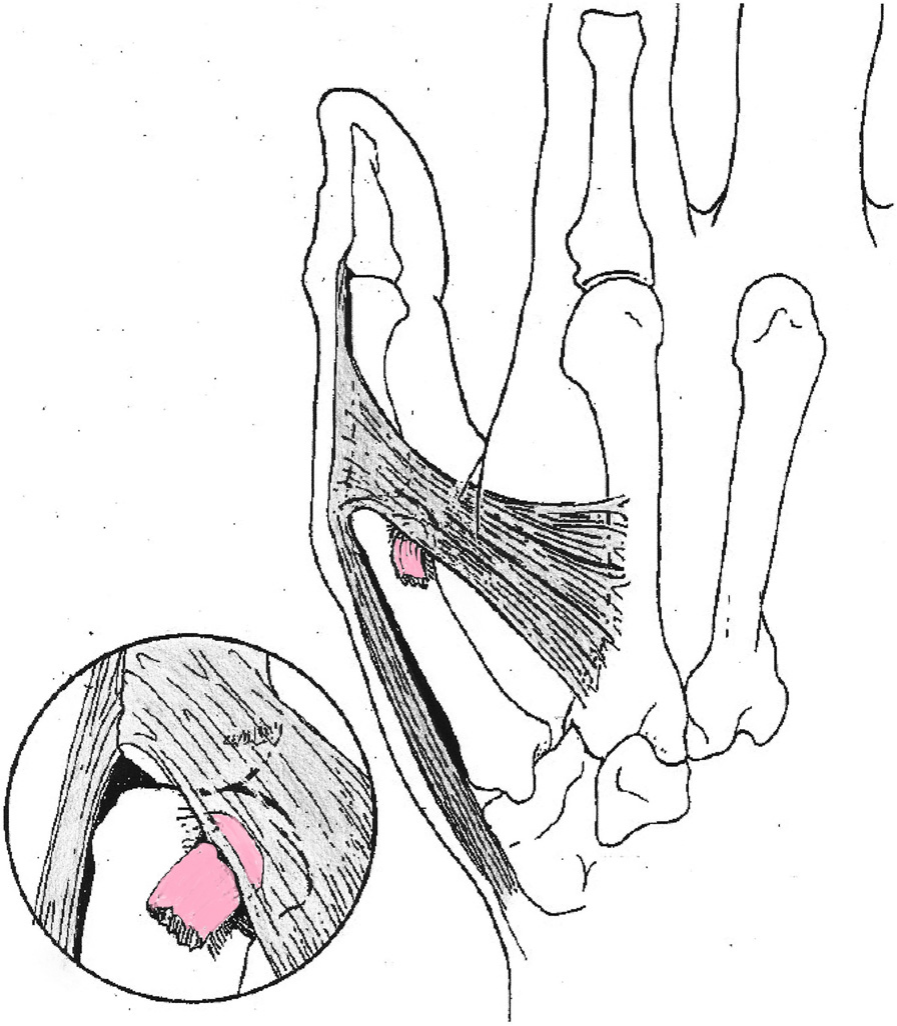
A Stener lesion.

The most important dynamic component is the adductor pollicis muscle. This muscle has its insertion onto the proximal phalanx partly superficial to and partly deeper than the UCL. This relationship is crucial to understanding how a Stener lesion can occur [6]. Most of the time, the distal end of the UCL ruptures. A Stener lesion occurs when this part gets stuck between the proximal edge of the still intact aponeurosis of the adductor (Figure 2). Because this aponeurosis stands between the UCL and the bone, it is thought that this injury cannot heal in this position. Stener lesions occur in 64% to 87% of all complete ruptures [6,7] and are usually treated by surgical repair.

If the MCP joint is in flexion, the PCL and the dorsal capsule are taut and therefore the most important stabilizers in that position. The reverse applies to the ACL and the volar plate, which are taut when the MCP is in extension [7]. This is important to know when testing the stability of the joint.

When laxity during testing is only seen with the MCP in flexion, an isolated PCL rupture is suggested. If this laxity is seen in flexion and extension, a complete rupture of the PCL and ACL is most likely.

## Review

### Physical examination

If the patient’s anamnesis suggests an injury of the UCL, this can be tested with a physical examination. Usually the patient has pain, swelling and a hematoma at the ulnar side of the MCP joint of the thumb. Sometimes a mass can be felt in that area, which suggests a Stener lesion; however, it is not pathognomonic [8].

**Figure 3:**
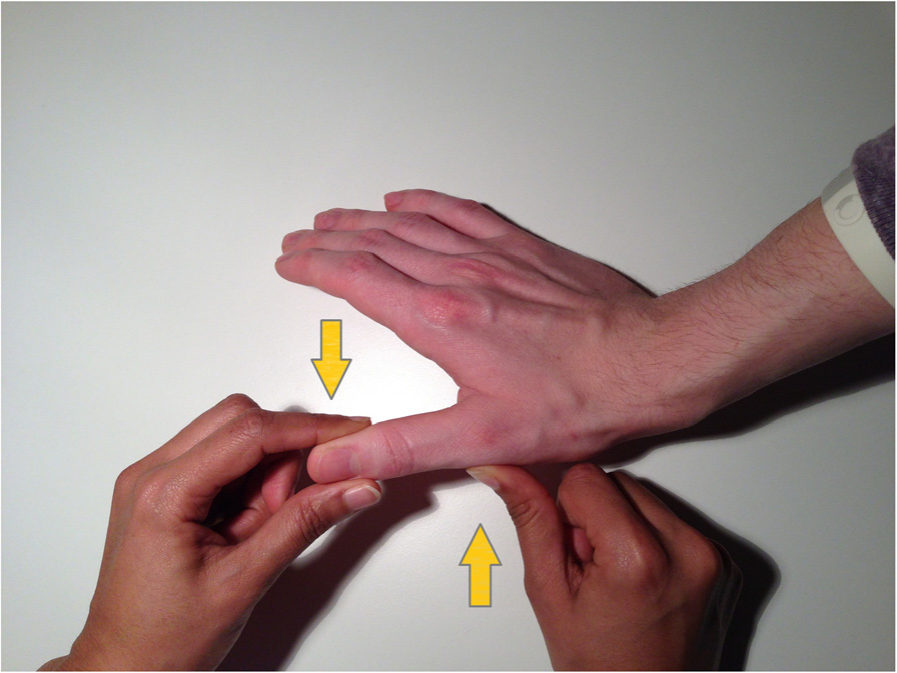
Testing of the UCL with MCP in extension.

**Figure 4:**
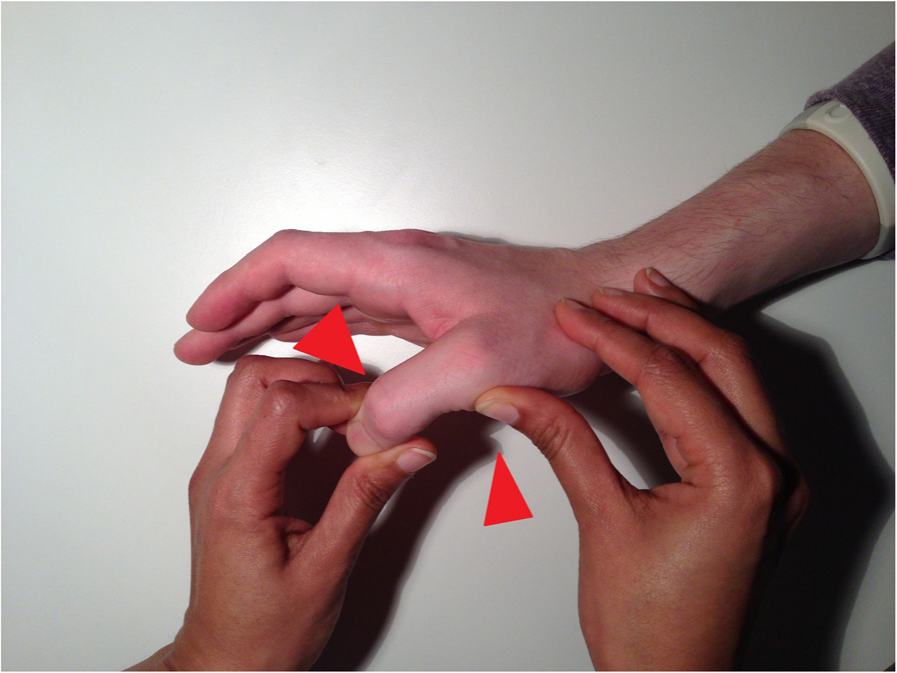
Testing of the UCL with MCP in flexion.

The UCL is tested by first holding the MCP in extension and applying valgus stress to the phalanx. The same is done with the MCP in 30 degrees of flexion. It is important that the thumb of the investigator is placed on the radial side of the MCP joint to apply counter pressure to prevent possible rotational effects (Figures 3 and 4).

It is difficult to say when a true laxity of the joint is seen, because the normal range of motion of the MCP joint differs per individual. In most of the literature the standard is more than 35 degrees during valgus stress and/or more than a 15 degrees difference compared to the contralateral side to diagnose a total rupture [7,9].

However, in a recent study [10] in which laxity in healthy test subjects was tested, it was found that 34% of all people have a more than 10-degree left-right difference in extension, and 12% had a difference of 15 degrees or more. In flexion this was seen in 22% and 3% of patients, respectively. The advice of Ritting et al. [8] in a recent review was that instead of holding on to a fixed degree limit, the absence of a firm endpoint during testing is a more reliable criterion when clinically diagnosing a complete rupture of the UCL. However, this can only be reliable when the investigator has enough clinical experience with testing the UCL.

Often the examination is too painful to perform and the results cannot be interpreted correctly because of an uncooperative patient. Performing the investigation under local anesthesia can be useful. A study by Cooper et al. [11] described how Oberst anesthesia (in which 1–2 ml of lidocaine is injected in the MCP joint on the ulnar and radial side) increases the clinical accuracy from 28% to 98% after an average of one week after the initial trauma. Sometimes the swelling during initial presentation can stand in the way of performing a reliable physical examination. In this case, one can decide to immobilize the hand and re-evaluate it after a week, with or without using Oberst anesthesia.

Only the difference between a partial and a total rupture can be diagnosed with a physical examination. A Stener lesion is a type of complete rupture that cannot be differentiated from a total rupture in which the UCL is still close to its insertion. As mentioned before, a swelling at the MCP does suggest a Stener lesion but is not specific for one. This difference can only be visualized by additional imaging or during surgery.

Some investigators have their reservations when testing the stability of the joint because they are worried about dislocating an avulsion fracture of the insertion of the UCL onto the proximal phalanx. Often the advice is to first make an X-ray and to test the hand clinically only when there is no avulsion fracture. However, if the initial trauma was insufficient to cause a dislocation, the thought is that the strength used to test the thumb clinically is not enough to cause one [12]. Also, in a study with cadavers [13], a Stener lesion could not be caused by a correct clinical examination.

### Imaging studies

**Figure 5:**
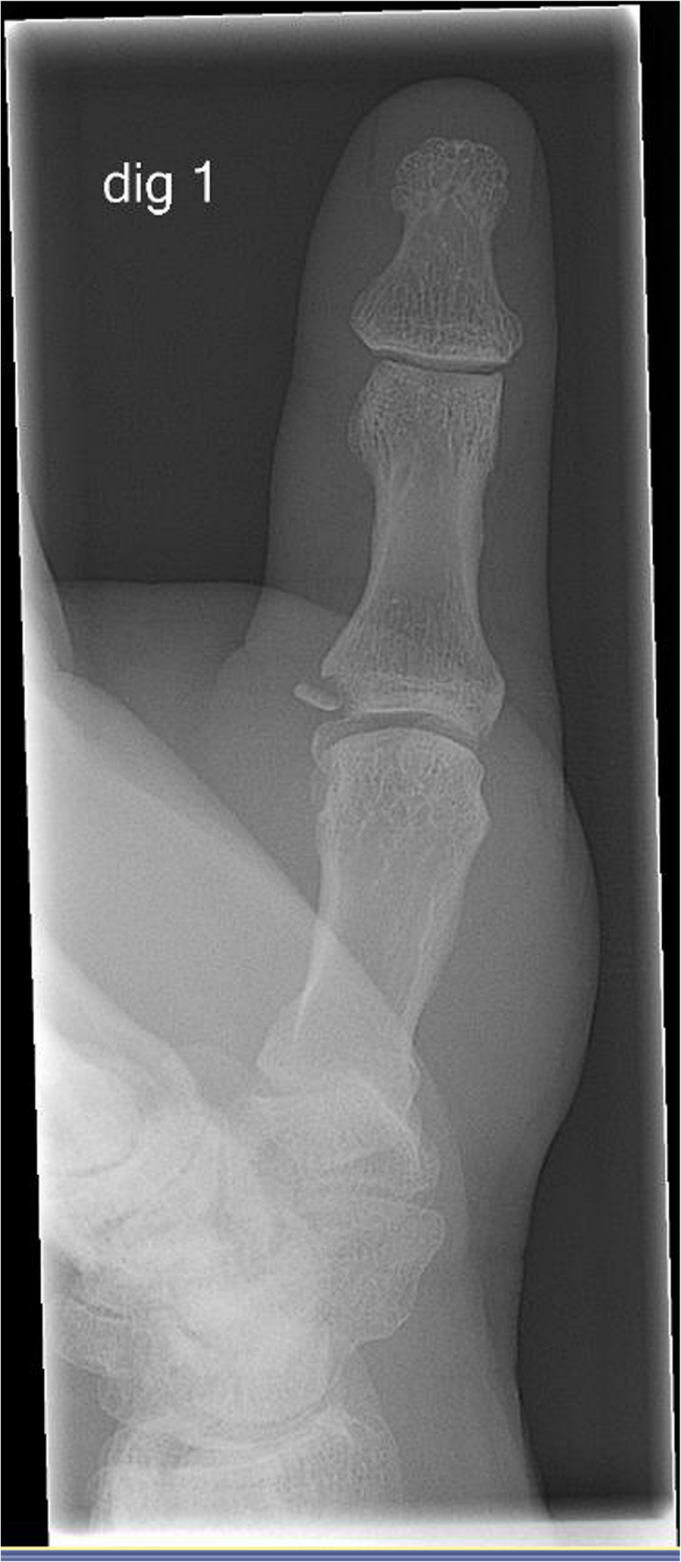
Radiograph of a bony skier’s thumb.

The first step in imaging studies is to make a plain radiograph in the AP and lateral direction to diagnose an avulsion fracture that is mostly located on the ulnar side of the proximal phalanx (Figure 5). A fragment is considered to be dislocated if it is displaced more than 1 mm or if it is malrotated. Stress radiographs are occasionally made to diagnose an UCL injury in patients who also have an avulsion fracture. Sometimes an avulsion fragment of the proximal phalanx can occur in a place where the UCL does not attach. A stress radiograph helps to distinguish between these types of injuries. It is difficult to standardize these results, and they are often ambiguous in interpretation. False-negative results of up to 25% of all patients have been found [14] in studies on the usefulness of this type of imaging. One of the issues with this type of imaging is that patients are often uncooperative because of pain. Opinions therefore still differ about whether this type of imaging is useful as a diagnostic tool.

If the plain radiograph shows no avulsion fragment but there is a clinical suspicion of skier’s thumb, further imaging can be performed by doing an ultrasound, CT, arthrogram or MRI. Which technique to use seems to be determined by the physician’s preference; there are no clear guidelines about this.

**Figure 6:**
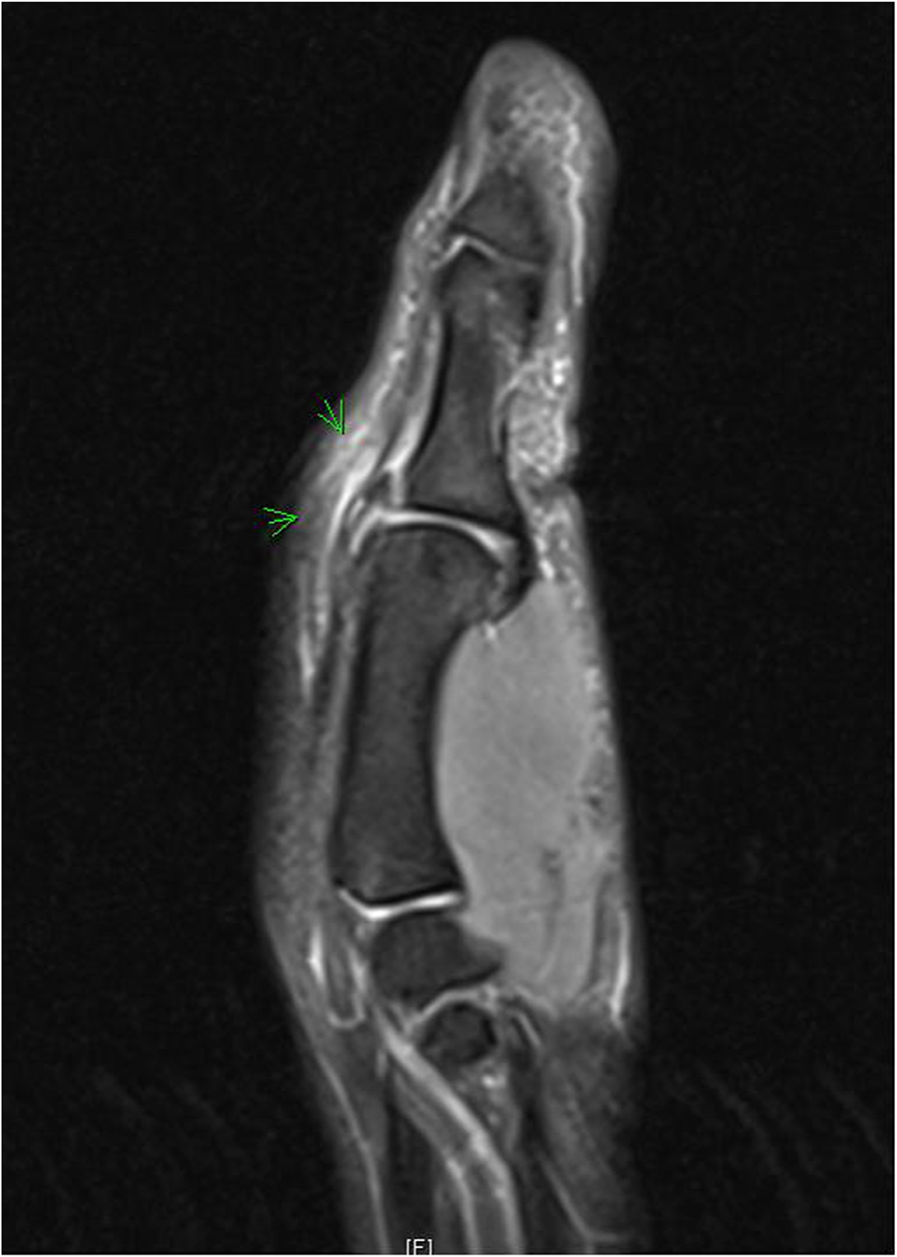
MRI of an old avulsion fracture of the UCL insertion.

MRI can be seen as a gold standard with a sensitivity of 96%-100% and specificity of 95-100% [15,16] (Figure 6). However, this is a very costly technique, often with long waiting lists. An alternative can be an ultrasound of the thumb. A recent study has shown that the ultrasound finding of absence of normal UCL fibers and presence of a heterogeneous mass proximal to the MCP joint is 100% accurate when diagnosing a complete UCL rupture [17]. Some limitations do apply however, such as the experience of the radiologist with ultrasounds of the musculoskeletal system. Also, the ultrasound cannot be performed later than 1 week after the initial trauma because shrinking of the torn ligament and scar tissue can be confounding when making a diagnosis. Our advice is, when necessary, to choose an additional imaging technique in conference with the radiologist in your own hospital. In our hospital, these ultrasounds are not performed because the resolution of the machine necessary to display the UCL correctly is insufficient.

### Treatment

The treatment of skier’s thumb is different for partial and a complete ruptures. Partial ruptures are treated conservatively. The MCP joint is immobilized, with the MCP fixed and the IP joint remaining free to prevent unnecessary stiffness. A navicular cast or brace is usually used.

The period of immobilization differs in the literature from 10 days to up to 6 weeks, and it seems to depend on the degree of laxity during the initial examination. Authors of a recent review on skier’s thumb agreed on a 4-week period [8]. An option is to adjust the length of this period to the clinical assessment of the patient’s pain. Physical therapy of the hand can be started afterwards.

Controversy also exists about treating a bony skier’s thumb without surgery. The literature however shows that if the MCP joint is stable during testing and there is no dislocation of the fragment, this injury can be treated conservatively without reason for concern [8,18].

If there is an unstable joint for which no firm endpoint is found during testing, and/or there is a dislocated, malrotated bony fragment or one that is more than one third of the joint surface, surgery is considered the best treatment. This also applies to Stener lesions because the general idea is that the UCL cannot heal if it is not in contact with its insertion, even though no evidence can be found in the literature to support this notion.

Also, no trials have even been set up to investigate whether a surgical intervention is really superior to a non-surgical treatment. Some small studies were carried out to see whether non-surgical treatment for a complete rupture could be equal to surgery. Landsman et al. [19] described 40 patients with a total rupture with and without a Stener lesion, which were all treated only by immobilization. Thirty-four patients were successfully treated this way; the other six still had complaints of instability and pain and underwent successfull operations. However, another study by Pichora et al. [20] reported that 3 of the 32 patients with total ruptures that were treated non-surgically had persisting complaints that could not be resolved with surgery. A recent review from 2012 [21] that, among others, compared multiple studies about treatment of a complete rupture concluded that a surgical intervention is preferred because they are known to have good and predictable results.

Different surgical techniques can be used. Which one applies depends on the anatomy of the lesion and can often only be decided upon during surgery. The UCL can be fixated with a suture anchor or with transosseous stitches. Small bone fragments can be removed; larger ones can be fixated with a Kirschner wire or a small screw. Results seem to be independent of the chosen technique, and successful recovery to the patient’s level before the initial trauma occurs in 90%-96% of all patients [8,21,22]. This means that the question remains whether the patients mentioned above (with persisting complaints after the first non-surgical and later surgical treatment) would have benefitted from initial surgical intervention.

Ideally, the operation takes place within 2 weeks; however, good results can still be achieved after 3–4 weeks [8]. Afterwards, a period of usually 6 weeks of immobilization is applied, after which a new radiograph is made and physical therapy of the hand can be started.

When the pain has subsided and the range of motion has completely returned, the hand can be completely used again. Usually this takes about 3 months [1].

Patients with worse outcomes are mostly patients with a delay in presentation [1,8]. When repaired in a timely manner, complications are rare. When they do occur, it usually concerns neuropraxia of the radial nerve that arises secondary to traction, swelling or stiffness. All are usually temporary in nature. Persistent instability is very rare. In the last 4 years we have only seen one patient with this complaint; however, he had a patient delay of several months.

Not enough information is available on the chances for recurrence with this type of injury.

## Conclusion

When evaluating a patient with skier’s thumb one must pay attention to a number of points. First, the laxity of the UCL needs to be tested in both flexion and extension of the MCP joint while counteracting possible rotational effects in the MCP joint. Because this examination can be quite painful during the initial presentation, applying Oberst anesthetics can be useful to gain a more reliable result. Instead of a fixed number of degrees, the absence of a firm endpoint seems to give a more accurate clinical diagnosis when determining a complete rupture.

If there is any doubt about the clinical diagnosis, MRI, which has a high sensitivity and specificity, is a useful tool. A cost-effective and timesaving alternative is an ultrasound, provided that there is enough experience at the hospital concerned.

Usually, partial ruptures are treated non-surgically and complete ruptures by surgical intervention. However, no higher level evidence can be found in the literature to support the notion that surgical treatment is superior to conservative treatment in case of a complete rupture.

Different techniques can be used when operating, which all seem to be equally effective. Complications are rare and mostly concern (temporary) neuropraxia of the radial nerve.

The recommended immobilization time is approximately 4–6 weeks, which is followed by a period of physical therapy. After an average of 3 months, the thumb can be fully used again.

### Consent

Written informed consent was obtained from the patient for the publication of this report and the accompanying image.

## Abbreviations

UCL: Ulnar collateral ligament; PCL: Proper collateral ligament; ACL: Accessory collateral ligament; MCP: Metacarpophalangeal.

## Competing interest

The authors declare that they have no competing interests.

## Authors’ contributions

MM carried out the literature research and drafted the manuscript. SR contributed in the design of the manuscript and revised the manuscript critically for intellectual and relevant content. Both authors read and approved the final manuscript.
